# Serum brain-derived neurotrophic factor as diagnosis clue for Alzheimer's disease: A cross-sectional observational study in the elderly

**DOI:** 10.3389/fpsyt.2023.1127658

**Published:** 2023-03-16

**Authors:** Yuanyuan Li, Jiao Chen, Hui Yu, Jiayu Ye, Chunxia Wang, Lingli Kong

**Affiliations:** ^1^Medical Department, Qingdao University, Qingdao, China; ^2^Department of Geriatric Psychiatry, Qingdao Mental Health Center, Qingdao, Shandong, China; ^3^School of Mental Health, Jining Medical University, Jining, Shandong, China

**Keywords:** Alzheimer's disease, BDNF, mBDNF, proBDNF, serum

## Abstract

**Objective:**

Brain-derived neurotrophic factor (BDNF) has not been validated as a diagnostic marker for Alzheimer's disease (AD). To provide a different perspective, this study aimed to evaluate the relationship between serum levels of mature BDNF (mBDNF) and precursor BDNF (proBDNF) in AD and to investigate whether serum BDNF levels or the ratio of mBDNF levels to proBDNF levels (M/P) could be a valuable biomarker for determining the risk of AD in elderly individuals.

**Method:**

A total of 126 subjects who met the inclusion criteria were assigned to either the AD group (*n* = 62) or the healthy control group (HC, *n* = 64) in this cross-sectional observationl study. Serum levels of mBDNF and proBDNF were measured using enzyme immunoassay kits. We analyzed the Mini-Mental State Examination (MMSE) scores from the two groups and examined the associations between AD and BDNF metabolism.

**Results:**

The serum concentration of proBDNF was significantly higher in ADs (4140.937 pg/ml) than in HCs (2606.943 pg/ml; *p* < 0.01). The MMSE significantly correlated with proBDNF (*p* < 0.01, r = −0.686) and M/P (*p* < 0.01, r = 0.595) in all subjects. To determine the risk for AD, the area under the receiver operating characteristic curve was calculated, which was 0.896 (95% confidence interval 0.844–0.949) for proBDNF and 0.901 (95% 0.850–0.953) for proBDNF and M/P combined.

**Conclusion:**

We observed a correlation between low serum proBDNF levels and higher MMSE scores in AD. The most effective diagnostic strategy was the combination of proBDNF and M/P, whereas mBDNF levels performed poorly when we evaluated the predictive model.

## 1. Introduction

Alzheimer's disease (AD), the most common form of dementia in elderly individuals, causes neuronal degeneration leading to cognitive deficits and altered behavior (1). Early stages of AD are characterized by difficulty consolidating and storing new memories. Later AD stages are characterized by gradual changes in cognitive abilities and behavior (2). Neurotrophic factors, including brain-derived neurotrophic factor (BDNF), can exhibit changes in their levels over time in AD. Neurons secrete BDNF in response to neuronal activity, which is critical for the formation of appropriate synaptic connections during embryological development and for learning and memory in adulthood (3). Meanwhile, BDNF is a critical factor in synaptic repair and plasticity, as well as in neuron survival, development, and maintenance (4). BDNF is the most abundant neurotrophin in the central nervous system (CNS), where it is involved in a number of important processes, including synaptic plasticity and neuron survival (5). Previous research on BDNF level's role in AD has yielded conflicting results. According to Mattson et al. (6), the decline in BDNF expression in primary brain regions affected by aging may be related to age-related impairments in cognitive function. BDNF levels in the central and peripheral nervous systems decrease with age (7), especially in older adults with mood disorders and cognitive impairment (8). Another study showed that sustained downregulation of BDNF in the serum and brain of AD patients appears to begin with the onset of the first clinical symptoms (9). Several studies have shown that BDNF expression is significantly reduced in the hippocampus, temporal, frontal (10), and parietal cortices (11) of AD patients compared to control subjects and that increased serum BDNF levels are associated with improved neuropsychological performance in healthy elderly subjects (12). In contrast to the control group, another study demonstrated a significant increase in serum BDNF levels in individuals with cognitive impairment (13). While several studies have shown that individuals with AD have lower (14) or higher (13) serum BDNF levels than healthy controls (HCs), other studies have shown no differences in BDNF levels between patients with AD and HCs (15). It remains uncertain whether BDNF has a causal function in neurodegeneration (16). Prohormone convertases such as furin and PC1/3 or plasminogen/plasmin and MMPs cleave proBDNF either intracellularly or extracellularly to release the mature form of 13.5 kDa after it is first formed as pre-proBDNF and converted into a proBDNF protein of 32 kDa (17, 18). The binding of proBDNF to the p75 Neurotrophin receptor causes neuronal cell death, decreased neuronal density, and negative regulation of genes for learning and memory, while the interaction between mBDNF and the TrkB receptor promotes neuronal survival, differentiation, and positive regulation of learning and memory (19, 20). Therefore, the relative amounts of BDNF isoforms might be crucial in differentiating psychiatric disorders. According to Peng's summary (21), in the early stages of AD, both proBDNF and mBDNF levels decrease and are associated with cognitive impairment scores, such as the Global Cognitive Score and the Mini-Mental State Examination Score (MMSE).

AD has a very insidious onset and is difficult to detect initially since there is no specific biomarker that can be clinically tested. Therefore, it is critical to find reliable biomarkers with a high degree of diagnostic sensitivity and specificity for AD. Degenerated neurons can block the conversion of proBDNF to mBDNF, resulting in an imbalance in the ratio of mBDNF levels to proBDNF levels (M/P) in neurodegenerative diseases (22). ProBDNF affects long-term potentiation and negatively regulates microglia complexity and neuronal density in the hippocampus *via* p75 neurotrophin receptor in mice (23). Furthermore, exposure of mature hippocampal neurons in culture to proBDNF has been shown to significantly decrease microglia neuronal density *via* caspase 3 (24). Consequently, a reduction in M/P may lead to a reduction in the microglia neuronal density of neurons in the hippocampus (25). Based on the above findings, M/P may reflect BDNF metabolism and serve as an indicator of the biological role of mBDNF and proBDNF in the brain (26). mBDNF and proBDNF, which are primarily produced or released by the brain, may also be produced in the periphery and CNS. The CNS is protected by the blood–brain barrier (BBB). By limiting permeability and ensuring selective flow of chemicals between the blood and the CNS, the BBB protects and regulates the CNS environment (27). It also prevents extracerebral blood from entering and causing damage to the parenchyma of the brain. It is worth noting that the BBB does not interfere with the ability of BDNF to move freely between the systemic and cerebral circulations (28). Because proBDNF and mBDNF can freely pass through the BBB, their serum concentrations can be considered equivalent to their actual concentrations in the CNS (29).

The primary objective of our study was to explore the potential correlation between mBDNF, proBDNF, and M/P and MMSE scores in patients with AD. Consistent with this hypothesis, we examined diagnostic value of these levels in AD patients.

## 2. Materials and methods

### 2.1. Subjects

Between March and October 2021, 126 subjects were recruited for the study, including 62 AD patients and 64 HCs. The study was approved by the local ethics committee and was conducted in accordance with the Declaration of Helsinki. Before participating in the study, all subjects provided written informed consent. Two psychiatrists clinically and psychologically assessed subjects who met the eligibility criteria.

The patient inclusion criteria for AD in the experimental group at Qingdao Mental Health Center included a diagnosis of AD according to the ICD-10 criteria, an age between 51 and 90 years, and an MMSE score of 18 or less, laboratory values that were within normal limits or not considered clinically significant by the investigator, a sufficient degree of vision and hearing to follow the testing procedures, amyloid-positive by amyloid positron emission tomography (PET) or cerebrospinal fluid (CSF) A1–42 detected to be eligible for the study, and not taking approved AD or mood-stabilizing medications or had been taking them for at least 3 months before study entry.

Patients with the following conditions were excluded: (1) severe medical conditions such as uncontrolled diabetes, chronic obstructive pulmonary disease or asthma, hematologic/oncologic disease, and B12 or folic acid deficiency; (2) comorbidity with primary psychiatric disorders (e.g., schizophrenia, major depression that began before the onset of AD) or neurologic disease (e.g., stroke, Parkinson's disease, seizure disorder, or head injury with loss of consciousness within the past year); (3) a known or suspected history of alcoholism or substance abuse; and (4) computed tomographic or magnetic resonance imaging evidence of focal parenchymal abnormalities.

The HC group consisted of subjects who visited the Shazikou Health Center for regular health checks that were matched to the subjects in the AD group by ethnicity, and place of birth. The healthy subjects were not related to each other or to individuals who had AD.

### 2.2. Clinical assessment

The MMSE, a brief 30-item questionnaire measuring the degree of cognitive impairment, has been used in research as a screening tool for dementia (30). In each survey taken by the study participants, the Chinese version of the MMSE was used to assess cognitive function. The MMSE was used to determine the severity of dementia in participants (31). At admission, two experienced psychiatrists independently performed MMSE assessments on the participants.

### 2.3. Blood collection and isolation of serum samples

One hundred and twenty-six subjects consented to blood sampling. Whole blood was drawn from each subject through venipuncture. To obtain the serum, the whole blood samples were allowed to clot at room temperature (RT) for 30 min and then centrifuged at 1,000 × g for 15 min at RT. The serum was then carefully collected and stored at−80°C until evaluated using ELISA.

### 2.4. Enzyme-linked immunosorbent assay

Serum levels of mBDNF and proBDNF were determined in serum using a commercial BDNF ELISA kit (Shanghai Bogoo Biotechnology Co., Ltd. Shanghai). This assay has high sensitivity and excellent specificity for detection of BDNF. No significant cross reaction with other soluble structural analogs. The ELISA was performed in duplicate with each serum sample according to the manufacturer's instructions. Optical density measurements (OD) were determined using a microplate reader (Mulltiskan MK3, Thermo Electron, USA) at a wavelength of 450 nm. Serum levels of mBDNF and proBDNF in test serum samples were estimated from the standard curve created using OD measurements. Laboratory personnel involved in the measurement of serum mBDNF and proBDNF levels were blinded to the health status of the subjects.

We simply coated the Nunc 96-well ELISA plate with the capture antibody (protein G-purified mouse anti-mBDNF monoclonal antibody, B34D10) and incubated it overnight at RT. After washing it three times with PBS, the plate was blocked with 3% BSA/PBS for 1 h at 37°C. The test serum samples and standards were properly diluted and added to the plate (100 μL/well) before incubation at 37°C for 1 h. The plate was read at 450 nm in a microplate reader after being incubated sequentially with detection antibody (2.5 pg/ml), streptavidin-HRP, and TMB substrate (model Sunrise, TECAN, Germany). Serum mBDNF and proBDNF concentrations were estimated as ng/ml of protein. Each plate contained an internal control serum sample.

### 2.5. Potential correlations

Based on previous studies (14), the two demographic variables age and years of education were selected as possible confounders for determining the association between serum levels and MMSE.

### 2.6. Statistical analysis

The results are presented as the mean ± SD or median and quartiles using IBM SPSS Statistics for Windows, version 26. The 95% confidence intervals (CI) for odds ratios were determined (ORs). The independent *t* test (two samples), Mann–Whitney U test, chi-square test, Fisher's exact test, or analysis of variance (ANOVA) were used to determine the statistical significance of differences between groups. Crude associations between variables and outcome measures were first assessed with simple linear regression analyses. After partial correlation analysis for potential confounding variables, multiple linear regression analyses were then performed (age and education). We also conducted a logistic regression analysis with odds ratios (ORs) and 95% confidence intervals (CIs) to assess the association between BDNF and AD in all subjects. Multiple linear regression analysis using age, MMSE scores, and years of education as the independent variables, and mBDNF, proBDNF, and M/P as the dependent variables was performed. To select the optimal model for predicting the occurrence of AD, discriminative ability was assessed by calculating the area under the receiver operating characteristic (ROC) curves. Optimal cutoff values for each biomarker were determined using the highest Youden index (sensitivity + specificity – 1). For all statistical tests, the significance level was set at *p* < 0.05.

## 3. Result

### 3.1. Demographic characteristics between AD patients and HCs

One hundred and twenty-six individuals signed up to participate in the study. Table 1 provides an overview of the demographic and clinical characteristics of the experimental groups. The subjects included 64 healthy individuals and 62 individuals with AD. Although the average age of the two groups differs by 4 years, there is no significant difference. Although age did not have a significant effect on serum mBDNF levels in HC, Table 2 shows that it had a significant effect on serum proBDNF levels and M/P in AD. As seen in Table 2, there was no significant association between serum levels of mBDNF, proBDNF, M/P, and years of education (AD, HC and all subjects). There were no significant differences in the ratio of females to males between the AD and HC groups (*p* = 0.598, Pearson chi-square test with two restrictions, Table 1). The male and female groups did not differ significantly in mBDNF levels, proBDNF levels, and M/P (*p* = 0.371, 0.855, and 0.276, respectively). Male subjects (mean ± SD = 362.11 ± 79.01 pg/ml) had slightly lower mBDNF levels than female subjects (mean ± SD = 386.02 ± 97.08 pg/ml), whereas proBDNF levels were almost equal in men (mean ± SD = 3362.48 ± 1275.86 pg/ml) and women (mean ± SD = 3361.12 ± 1264.77 pg/ml).

**Table 1:** Participant demographic and clinical features.

	**AD (*N* = 62)**	**HC (*N* = 64)**	**t or χ2**	***P***
Age (years)	73.39 ± 8.153	69.38 ± 8.766	−2.658	0.009
Male/Female	31/31	29/35	0.277	0.598
Education (years)	7.11 ± 4.784	8.09 ± 3.486	1.312	0.192
Duration of AD (years)	4.25	-	-	-
MMSE score (max = 30)	9.77 ± 5.252	27.5	17.515	0.000

Mean ± S.D., or Median. HC, healthy controls; AD, Alzheimer's disease; MMSE, Mini-Mental State Examination.

**Table 2:** Correlation analysis among mBDNF, proBDNF, M/P ratio, demographic data, and clinical characteristics.

	**mBDNF**		**proBDNF**		**M/P**	
	***p***	***r***	***p***	***r***	***p***	***r***
Age (AD)	0.059	−0.241	0.047^*^	0.253	0.007^**^	−0.342
Age (HC)	0.360	−0.116	0.221	−0.155	0.802	−0.032
Age (all subjects)	0.020^*^	−0.207	0.032^*^	0.191	0.001^**^	−0.289
Education (AD)	0.607	−0.067	0.486	0.090	0.380	−0.113
Education (HC)	0.924	0.012	0.225	−0.154	0.376	0.112
Education (all subjects)	0.973	0.003	0.258	−0.102	0.401	0.075
Duration (AD)	0.175	−0.175	0.001^**^	0.407	0.000^***^	−0.429
After adjustment for age and education	0.884	−0.019	0.164	0.182	0.053	−0.251
MMSE (AD)	0.814	0.030	0.000^****^	−0.494	0.001^**^	0.425
After adjustment for age and education	0.558	−0.077	0.000^***^	−0.437	0.009^**^	0.333
MMSE (HC)	0.624	−0.062	0.277	−0.138	0.762	0.039
After adjustment for age and education	0.748	−0.042	0.910	0.015	0.737	−0.043
MMSE (all subjects)	0.180	0.120	0.000^****^	−0.686	0.000^****^	0.595
After adjustment for age and education	0.271	0.100	0.000^****^	−0.651	0.000^****^	−0.530

p-values were assessed using the Pearson correlation coefficient or Spearman rank correlation analysis. Significance was indicated with an ^*^when p < 0.05, ^**^when p < 0.01, ^***^when p < 0.001, and ^****^when p < 0.0001. AD, Alzheimer's disease; mBDNF, mature brain-derived neurotrophic factor; proBDNF, the precursor of brain-derived neurotrophic factor; M/P, the ratio of mBDNF levels to proBDNF levels; HC, healthy control; MMSE, Mini-Mental State Examination.

We conducted a logistic regression analysis with odds ratios (ORs) and 95% confidence intervals (CIs) to assess the association between features and AD in all subjects. The final logistic models 1 and 2 have statistical significance. Model 1 explained the 93.5% variation (Nagelkerke *R*^2^) of AD and correctly classified 94.4% of the subjects. Model 2 explained the 60.7% variation (Nagelkerke *R*^2^) of AD and correctly classified 82.5% of the subjects. Among the six predictors included in the model, age, gender, education level and mBDNF were not statistically significant (see Table 3). proBDNF has significant significance in both models, with and without MMSE scores (shows in Table 3).

**Table 3:** Odds ratios of AD according to different features in all subjects.

**Variables**	**Model 1** ^**a**^			**Model 2** ^**b**^		
	**OR**	**95% CI**	***p*-value**	**OR**	**95% CI**	***p*-value**
Age	0.948	(0.823–1.092)	0.458	1.030	(0.974–1.089)	0.306
Sex	1.263	(0.040–39.930)	0.895	0.920	(0.326–2.597)	0.874
Education	1.310	(0.755–2.275)	0.337	0.943	(0.834–1.066)	0.349
MMSE scores	0.317	(0.133–0.754)	0.009^**^	-	-	-
mBDNF	0.998	(0.984–1.012)	0.750	0.997	(0.991–1.002)	0.227
proBDNF	1.003	(1.000–1.005)	0.041^*^	1.002	(1.001–1.003)	0.000^****^

^*a*^Model 1was with MMSE scores. ^*b*^Model 2 was with MMSE scores removed. P-values were the result of multivariate logistic regression analysis. OR, odds ratio; CI, confidence interval; AD, Alzheimer's disease; mBDNF, mature brain-derived neurotrophic factor; proBDNF, the precursor of brain-derived neurotrophic factor; MMSE, Mini-Mental State Examination. Significance was indicated with an ^*^when p < 0.05, ^**^when p < 0.01, and ^****^when p < 0.0001.

We also conducted a logistic regression analysis with assess the association between three BDNF parameters (mBDNF, proBDNF and M/P) and the presence in all subjects and the three logistic regression models demonstrated good performance with overall accuracy of 59.5, 81, and 74.6% separately. The results showed that mBDNF had a significant negative effect on the outcome (coefficient = −0.005, SE = 0.002, *p* = 0.022) and M/P also had a significant negative effect on the outcome (coefficient = −37.428, SE = 7.085, *p* < 0.000) while M/P had a significant positive effect on the outcome (coefficient = 0.002, SE = 0.000, *p* < 0.000).

### 3.2. Serum mBDNF and proBDNF levels in AD patients and HCs

In all subjects, serum proBDNF levels were significantly higher (*p* < 0.0001) in AD patients (mean ± SD = 4140.94 ± 665.11 pg/ml) than in HCs (mean ± SD =2606.94 ± 1267.78 pg/ml), whereas there was no significant difference in serum mBDNF levels (*p* = 0.063).

**Figure 1:**
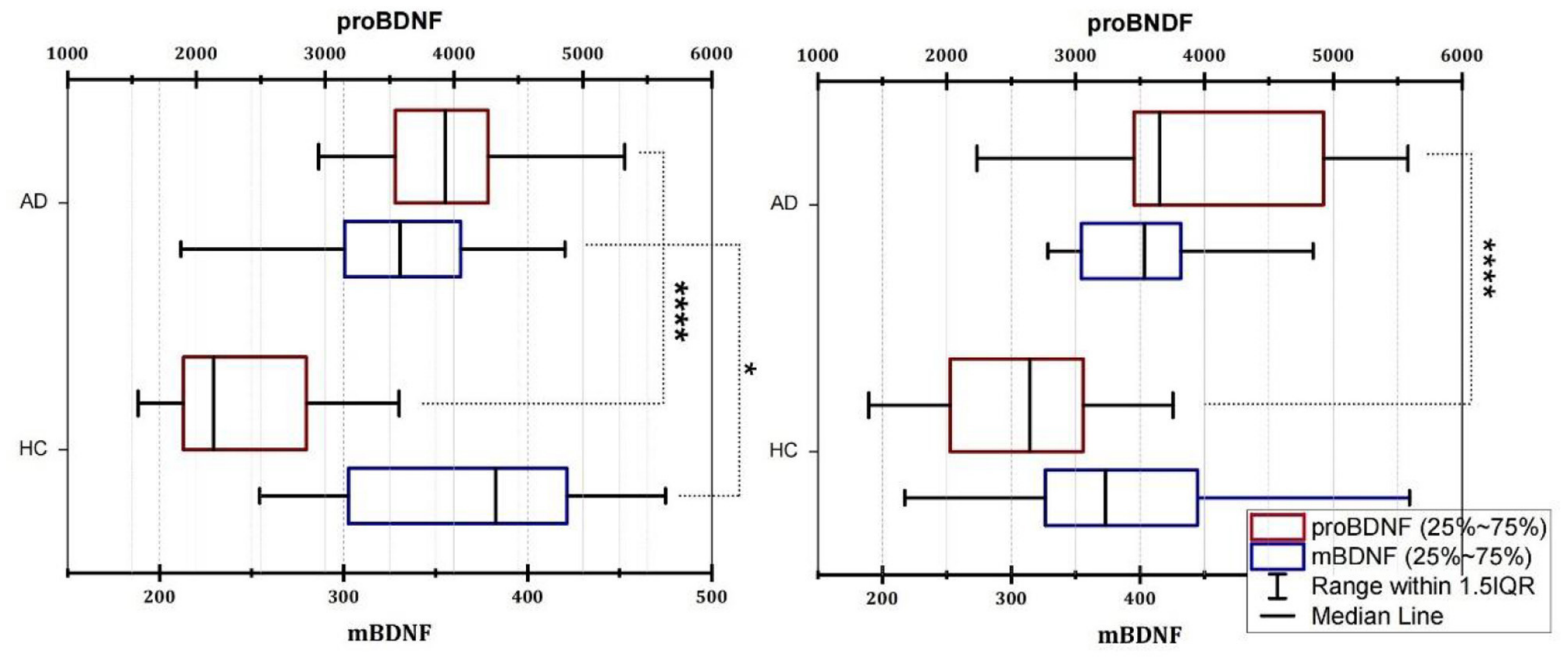
Box plot of the serum levels of mBDNF and proBDNF (ELISA) in group age 61–70 **(Left)** and group 71–80 **(Right)**. AD, Alzheimer's disease; HC, healthy control; mBDNF, mature brain-derived neurotrophic factor; proBDNF, the precursor of brain-derived neurotrophic factor. The horizontal lines in the middle of the boxes represent the median and interquartile range [IQR] and the data were analyzed using the Mann–Whitney (M-W) U test or *t*-test. Control, healthy controls; AD, Alzheimer's disease. Significance was indicated with an *when *p* < 0.05, and ****when *p* < 0.0001.

Because the age distribution of AD and HC subjects in this study is not consistent, we grouped them by age and divided participants into four age groups: 51–60, 61–70, 71–80, and 81–90 years old. However, due to the small number of people in the 51–60 and 81–90 age groups, we used the 61–70 age group and the 71–80 age group to explore the distribution of mBDNF and proBDNF in AD and HC, and to determine whether there were differences between them. Figure 1 shows the differences in mBDNF and proBDNF levels between the HC and AD patients in the two age-matched groups. The box plot revealed a clear difference in the distribution of difference (*p* < 0.0001) in the distribution of proBDNF between AD and HC in the 61–70 and 71–80 age groups, as indicated by the box plot (Figure 1). While age group had a significant effect on mBDNF levels in group 61–70 years old, the magnitude of this effect varied by age group. Specifically, in the younger age group, mBDNF levels were significantly different between the AD and HC (Figure 1, Left), whereas in the older age group (Figure 1, Right), there was no significant difference in mBDNF levels. These findings suggest that the effect of age on mBDNF levels may depend on the age group being considered. The 25–75% range of mBDNF as having a similar and even overlapping distribution in both age groups, whereas the 25–75% range of proBDNF is quite different and does not overlap. The median of proBDNF in AD was significantly higher than that of HC in both groups, as evidenced by the non-overlapping interquartile ranges (Figure 1).

### 3.3. Correlation analysis

**Figure 2:**
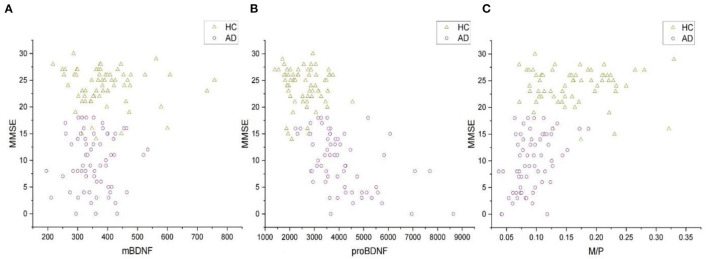
The correlations of MMSE and serum mBDNF, proBDNF, and M/P levels in all subjects. **(A)** The correlation between mBDNF and MMSE. **(B)** The correlation between proBDNF and MMSE. **(C)** The correlation between M/P and MMSE. All the data were analyzed by Spearman's correlation test. mBDNF, mature brain-derived neurotrophic factor; proBDNF, the precursor of brain-derived neurotrophic factor; M/P, the ratio of mBDNF levels to proBDNF levels; MMSE, Mini-Mental State Examination.

Increased serum proBDNF levels correlate with the severity of cognitive impairment (Figure 2B), which is associated with a decrease in M/P (Figure 2C). The mean MMSE value in the AD group was 9.77 ± 5.25, whereas it was 23.80 ± 3.54 in the HC group (Table 1). MMSE scores were positively related to M/P levels (*p* < 0.0001, *r* = 0.595) but negatively correlated with proBDNF serum levels (*p* < 0.0001, *r* = −0.686), as shown in Figures 2B, C, respectively. This correlation remained significant after adjustment for age and years of education (see Table 2). In HCs, age, years of education, and MMSE did not correlate with proBDNF levels and M/P. mBDNF level was not related to age, years of education, or MMSE, although there was a marginal correlation between mBDNF levels and age in the AD group (r = −0.241, *p* = 0.059) (Table 2). After adjustment for age and years of education using multiple linear regression, the significant correlation between MMSE in AD patients and proBDNF and M/P remained, although somewhat weaker; the significant correlations between time and proBDNF and M/P did not remain the same. Increased proBDNF and decreasing serum M/P levels are associated with aging.

Our results of multiple linear regression analysis showed that MMSE scores were significant predictors of proBDNF and M/P levels (*R*^2^ = 0.451, *p* < 0.0001, β = −101.728 and *R*^2^ = 0.330, *p* < 0.0001, β = 0.004, respectively), but not of mBDNF (*R*^2^ = 0.088, *p* = 0.271).

### 3.4. Best model to predict AD

**Figure 3:**
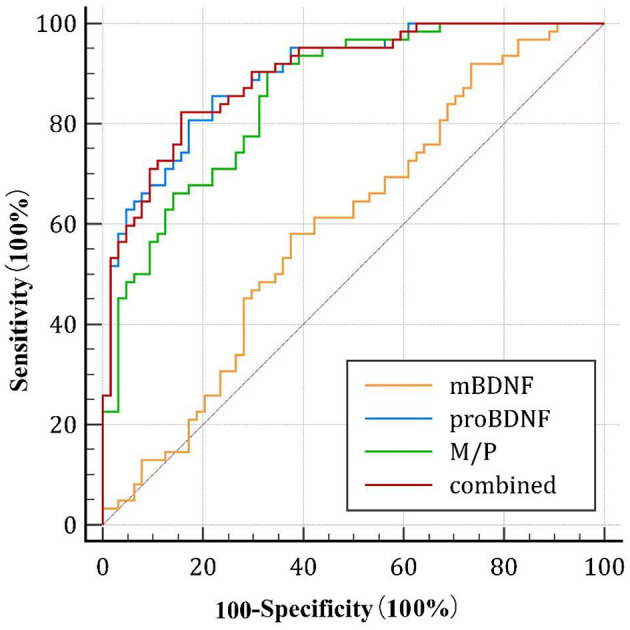
Diagnostic value of the serum mBDNF, proBDNF, and M/P levels. ROC, receiver operating characteristic; mBDNF, mature brain-derived neurotrophic factor; proBDNF, the precursor of brain-derived neurotrophic factor; M/P, the ratio of mBDNF levels to proBDNF levels.

As shown in Figure 3, the area under the curve (AUC) of serum mBDNF levels, proBDNF levels, and M/P was 0.596 (CI: 0.496–0.696), 0.896 (CI: 0.844–0.949) and 0.856 (CI:0.793–0.920), respectively. ProBDNF levels and M/P combined were the most accurate predictors for the development of AD in patients, with an AUC of 0.901 (CI: 0.850–0.953), as revealed by using logistic regression analysis. Table 4 summarizes the diagnosis for all three serum levels.

**Table 4:** Diagnostic value of the serum mBDNF levels, proBDNFlevels, and M/P.

	**mBDNF**	**proBDNF**	**M/P**	**proBDNF and M/P combined**
AUC (95% CI)	0.596 (0.496–0.696)	0.896 (0.844–0.949)	0.856 (0.793–0.920)	0.901 (0.850–0.953)
Cutoff value	≤ 360.505	>3073.449	≤ 0.127	>0.573
Sensitivity, %	58.06	85.48	91.94	79.03
Specificity, %	62.50	78.12	65.62	85.94
*p*	0.059	0.000	0.000	0.000

AUC, area under the curve; mBDNF, mature brain-derived neurotrophic factor; proBDNF, the precursor of brain-derived neurotrophic factor; M/P, the ratio of mBDNF levels to proBDNF levels.

## 4. Discussion

In the absence of a specific biomarker for AD, we attempted to build a model for predicting AD using proBDNF levels and M/P. However, the literature on BDNF levels in AD is inconsistent. Previous studies of this type have mostly focused on serum BDNF levels. There are some clinical reports on proBDNF levels, mBDNF levels, or M/P in other neurodegenerative diseases (22), but to our knowledge, none on M/P in AD patients.

In this study, we determined the serum levels of proBDNF and mBDNF in AD patients and HCs using an immunoenzymatic assay and estimated their ratio to better understand these issues. We found that M/P was significantly reduced in AD patients compared with control subjects, and that M/P was significantly related to MMSE. This is in stark contrast to the results of Peng et al. (21), which showed a positive association between brain mBDNF and proBDNF levels and MMSE scores. Our results suggest that only proBDNF levels and M/P were associated with MMSE scores in all subjects, whereas mBDNF level was not. The results also showed that mBDNF levels were lower in the AD group than in the control group. However, proBDNF levels were higher in the AD group than in the control group. This is a significant difference and is consistent with some results from previous literature.

This study is unique in that the combination of proBDNF and M/P works better as a diagnostic marker for AD than proBDNF levels alone. At both initial diagnosis and follow-up, the association of proBDNF levels with MMSEs in AD patients was greater than that of M/P. Both proBDNF level, which has been recommended as a viable diagnostic biomarker for AD, and M/P were shown to be strongly associated with MMSEs in AD patients, although their correlation was inconclusive. Based on our results, we observed that individuals with AD have higher levels of pro BDNF compared to controls, as illustrated in Figure 1 and a reduction in the M/P in AD patients, as shown in Figure 2. These findings may suggest that the decreased conversion of proBDNF to mBDNF in AD patients contributes to the observed differences. We also did not reach the same conclusion (as we had expected) as the aforementioned study did regarding the correlation between higher education and better MMSE scores. In a German study, researchers demonstrated a negative association between blood BDNF levels and age in healthy elderly individuals (*n* = 259) (32). However, our results suggest that age was not associated with mBDNF or proBDNF levels in HCs (*n* = 64), but our study found that increasing age was associated with increased proBDNF levels and decreased M/P in AD patients. According to one study, there was no association between serum BDNF levels and age in the AD, MCI, and control groups in the North Indian population (33).

Thus, the discrepancies between our data and those in the abovementioned study could be explained by the smaller sample size of their study and geographic differences between their study and our study. While the current study did not analyze the interaction effect of age and proBDNF levels, it is worth noting that aging is a well-established risk factor for the development of AD. Therefore, the observed association between proBDNF levels and age may suggest that age-related changes in proBDNF play a role in the development of cognitive impairment in aging and AD. However, further research is needed to explore this potential interaction effect.

Male subjects had slightly higher mBDNF levels than female subjects, whereas proBDNF levels were almost the same in men and women. which contradicts previous findings that women have higher blood BDNF levels than men according to the results of biochemical studies (34). In a recent study (35), researchers discovered a female-specific genetic association between rs6265 and AD and sex-specific BDNF mRNA expression in brain tissue from AD patients. In addition, the results showed that BDNF might be a female-specific AD risk gene. According to researchers in the field, biomarkers could help explain the etiology of mental illness, be used to validate diagnoses, identify at-risk individuals, and measure the severity of a patient's illness. Because of their high sensitivity, specificity, and prognostic value, it is safe to use these biomarkers in this way. The most common clinical test used to diagnose many diseases is peripheral blood testing, and blood appears to be a convenient source of metabolic data because of its comparative ease of collection.

Clearly, maintaining healthy M/P levels is critical in AD. The huge increase in proBDNF levels has created several unintended consequences. Previous studies have shown that BDNF levels are not stable in people with mood disorders (36). Decreased BDNF levels have been linked to the development of AD (35). AD patients have a significant decrease in BDNF expression in the hippocampus, temporal and frontal cortices (37).

In the early stages of AD, serum BDNF levels can shift from increased to decreased according to studies by Laske et al. (38). These findings could be explained by an increase in BDNF level due to a compensatory mechanism in the brain (39). Apart from this, according to Angelucci et al. (40), BDNF levels were significantly higher in MCI and AD patients than in HCs, regardless of the severity of the disease.

Decreased BDNF levels may precede or follow the onset of neurodegenerative diseases. Concentration measurements at different time points may be helpful. The human hippocampus, amygdala, cerebellum, and cerebral cortices contain large amounts of BDNF, with hippocampal neurons having the highest BDNF levels (41, 42). Saturable transport systems provide a high capacity for delivery of intact BDNF across the blood–brain barrier, according to Pan et al. (43). Megakaryocytes and platelets are the main sources of BDNF production in the human body. However, these cells lack nuclei that enable them to synthesize BDNF (44). Fujimura et al. (45) discovered that the BDNF content in serum was equivalent to that of washed platelet lysates, suggesting that human platelets contain large amounts of BDNF protein; moreover, human serum contains much more BDNF than plasma because of platelet degranulation during the coagulation process. More than 100 times more BDNF was found in serum than in plasma (46). The low concentration of BDNF in CSF compared to serum could indicate BDNF's rapid metabolism in the plasma (47). In clinical practice, we consider blood levels of BDNF to be more reliable than CSF levels. Moreover, there is a high correlation between BDNF concentrations in the blood and brain circulations (48). Our study has some shortcomings that need to be addressed. One of the limitations of this study is that we did not include sex-matched controls, which could have affected the generalizability of our results. While we did not observe significant differences in mean age between our AD and control groups, a lack of age-matching could have introduced confounding variables. Therefore, future studies should include age-sex-matched controls to improve the robustness of the results. Additionally, there could be other unmeasured variables that were associated with sex that could have influenced the results. We believe that certain unobserved variables contribute to some of the differences in BDNF levels among patients, e.g., Met/Val homozygous carriers of the BDNF gene. However, a meta-analysis did not reveal conclusive evidence of cognitive deficits associated with this polymorphism (41). It is challenging to validate blood BDNF levels reported in clinical trials because of unknown confounders, variable outcomes dependent on patient age and sex (49), and miscalculation of BDNF levels in studies using parametric testing with small sample numbers (32). It is possible that the BDNF gene has additional polymorphisms that contribute to AD pathogenesis. The difference in blood BDNF concentrations among other populations may be attributed to differences in population recruitment since all our patients were recruited from eastern China, limiting population diversity. Larger independent samples at polymorphism locations within and near the gene are necessary to test whether the BDNF gene is linked to AD. Future research into modulating BDNF signaling may help reduce the risk of neurodegenerative disorders. Smoking, physical activity, body mass index, and serum cytokine levels were not considered in this study. Confounding research examining the relationship between BDNF serum levels and neurodegenerative illness may be caused by any or all these factors. These constraints should be considered in future research.

The body's BDNF levels may be affected by a variety of medications (50). AChEIs were administered to some of the patients in our study, which may have affected their blood BDNF levels, necessitating more research to assess their effects. To further characterize BDNF's role as a biomarker for AD, we need to better understand the alterations in the three versions of the protein. Future studies with larger and more diverse samples and controlled psychopharmacological states of the patients may be examined to replicate these findings and explore the underlying mechanisms of the observed associations.

## 5. Conclusion

In conclusion, proBDNF levels may be utilized as a biomarker for AD and are more effective than M/P at predicting the presence of AD. However, further information is required to make a conclusive decision while our study supports the hypothesis of a correlation between BDNF and AD presence, the retrospective, cross-sectional study design may limit the generalizability of our findings. Prospective studies should be conducted in the future to determine whether serum proBDNF levels and M/P can be used to predict the risk of AD.

## Data availability statement

The raw data supporting the conclusions of this article will be made available by the authors, without undue reservation.

## Ethics statement

The studies involving human participants were reviewed and approved by Qingdao Mental Health Center. The patients/participants provided their written informed consent to participate in this study.

## Author contributions

YL: conceptualization, methodology, data curation, and writing-original draft. JC and HY: formal analysis and writing-original draft. JY: data curation. CW: resources, supervision, and project administration. LK: conceptualization, resources, supervision, writing—review and editing, and project administration. All authors contributed to the article and approved the submitted version.
